# Uterine scar rupture at the site of the placenta accreta presenting as a case of sudden death

**DOI:** 10.4322/acr.2020.226

**Published:** 2020-12-08

**Authors:** Swapnil Prabhakar Akhade, Pankaj Suresh Ghormade, Ajay Bhengra, Krishnadutt Chavali, Nighat Hussain

**Affiliations:** 1 All India Institute of Medical Sciences, Department of Forensic Medicine and Toxicology, Raipur, Chhattisgarh, India; 2 Hazaribagh Medical College, Department of Forensic Medicine and Toxicology, Hazaribagh, Jharkhand, India; 3 All India Institute of Medical Sciences, Department of Pathology, Raipur, Chhattisgarh, India

**Keywords:** Cesarean Section, Maternal Death, Placenta Accreta, Pregnancy Complications, Uterine Rupture

## Abstract

Uterine rupture during pregnancy is a known complication of placenta accreta. This paper presents a case of sudden maternal death in the 27^th^ week of gestation due to a ruptured uterine scar at the site of placenta accreta with a short inter-pregnancy period of 6 months with previous two C-sections. Autopsy findings revealed a massive hemoperitoneum and a thinned out anterolateral uterine wall. Internal examination revealed clotted and fluid blood in the peritoneal cavity with rupture of the anterior uterine wall at the site of the placenta accreta in a healed cesarean section scar. Placenta accreta is a rare complication of pregnancy. However, it is becoming more frequent and a significant risk factor with the increasing rate of C-section.

## INTRODUCTION

As per World Health Organization, maternal death is the death of a woman while pregnant or within 42 days of termination of pregnancy, irrespective of the duration and site of the pregnancy, from any cause related to or aggravated by the pregnancy or its management but not from accidental or incidental causes.^1^ A high-risk pregnancy is one in which maternal environment or past reproductive performance poses a risk to fetal and/or maternal health.^2^ We report one such registered antenatal case of high-risk pregnancy admitted to our hospital for investigation and observation for placenta previa. Unfortunately, the patient left the hospital against the medical advice to be brought back four days later in a critical condition and eventually died.

## CASE REPORT

A 24-year-old multigravida, with approximately 27 weeks of gestation presented to the Emergency Department of the tertiary care hospital with complaints of a dull aching abdominal pain of 2-hour duration progressing to severe pain for an hour. She was a registered antenatal case in her fifth pregnancy (G_5_P_2_L_2_A_2_) with both her children delivered through lower segment cesarean sections (LSCS). As per her medical records, she had delivered her last child by LSCS 14 months ago, the indication being tenderness of the previous LSCS scar. Her children, one of 14 months and the other five years old, were healthy. She was a known case of hypothyroidism under medication.

During the current pregnancy, a routine antenatal ultrasound performed at about 24 weeks of gestation, showed findings of placenta previa and features consistent with a placenta accreta with intact amniotic membranes beyond the contour of the lower uterine segment. There was no sign suggestive of placental invasion of the bowel or urinary bladder.

The patient had visited our hospital for routine ANC check-up four days before her death. The couple had been counseled and advised by the treating obstetrician for hospitalization to further diagnostically evaluate the placenta previa. However, they refused admission and left against the medical advice citing family responsibilities, the proximity of residence to hospital, and favorable outcome with similar complaints in the previous pregnancy.

After four days, she was brought to the hospital in a critical condition with no palpable pulse and unrecordable blood pressure. Resuscitation attempts were unsuccessful. Fetal demise was also confirmed through absent fetal movements, and absent fetal heart sounds. The lady was declared dead, and as per prevalent rules, a medicolegal autopsy advised as a case of sudden death without a diagnosis for her immediate condition.

## AUTOPSY FINDINGS

**Figure 1:**
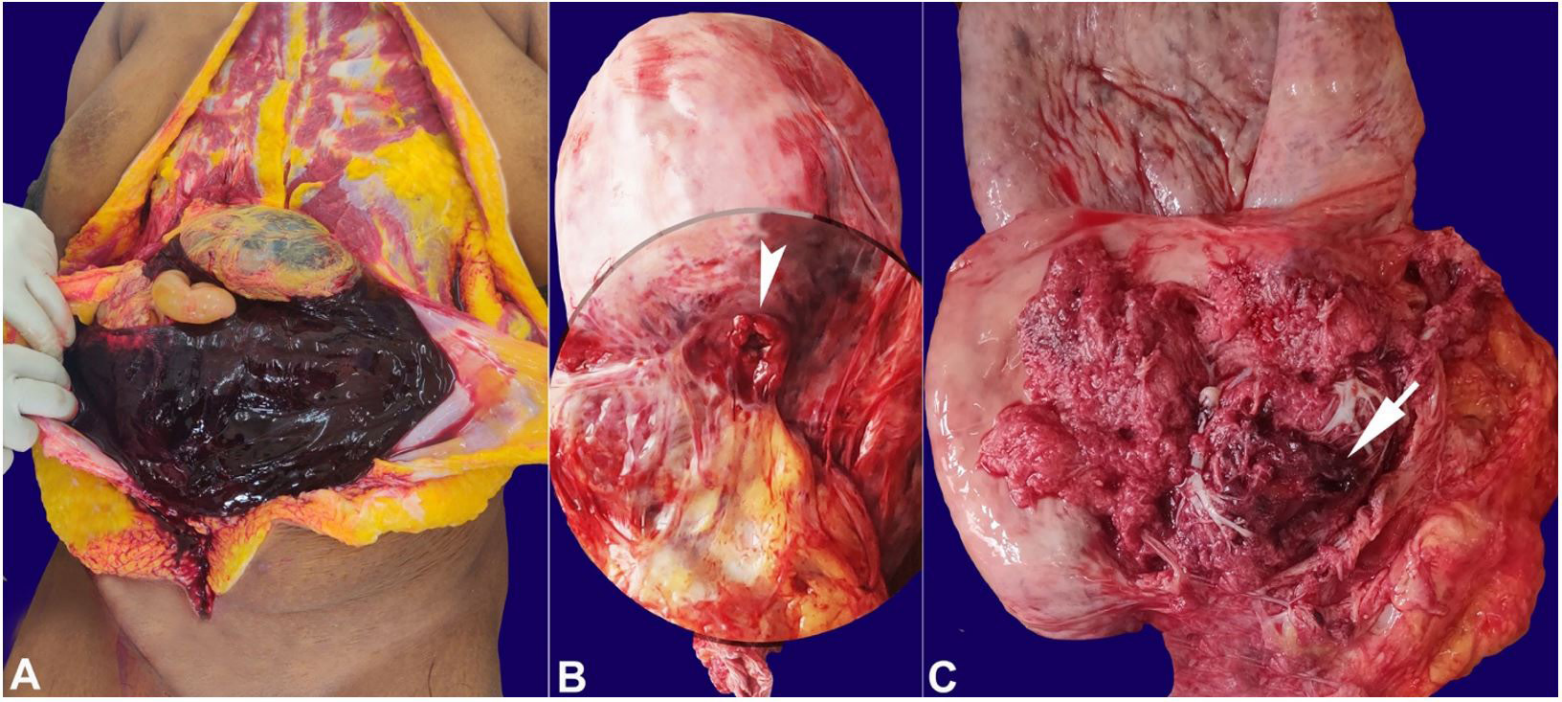
Gross view of the autopsy findings: **A –** hematoma in the peritoneal cavity, **B –** gravid uterus of 30cm x 18 cm with the thickness of 1 cm at the level of the fundus. The inferior aspect of the anterior wall, along the previous uterine scar, shows a uterine rupture of 1.2 cm in diameter (arrowhead), **C –**endometrial surface showing the invasion of the placenta into the myometrium, and serosa with hemorrhage (arrow).

At autopsy, on external examination, the abdominal girth at umbilicus was 85 cm. Generalized pallor was evident with pale conjunctiva and faint post-mortem lividity. On internal examination, all organs were pale with a collection of two-and-a-half liters of clotted and fluid blood in the peritoneal cavity (Figure 1A).

There was a perforation of the uterine wall, of 1.2 cm diameter, in the lower-left anterior wall of the uterus, at the site of LSCS scar (Figure 1B). The uterine wall around the perforation showed the placenta’s invasion through the myometrium up to the serosa, which was tightly adherent and inseparable from the uterine wall (Figure 1C).

**Figure 2:**
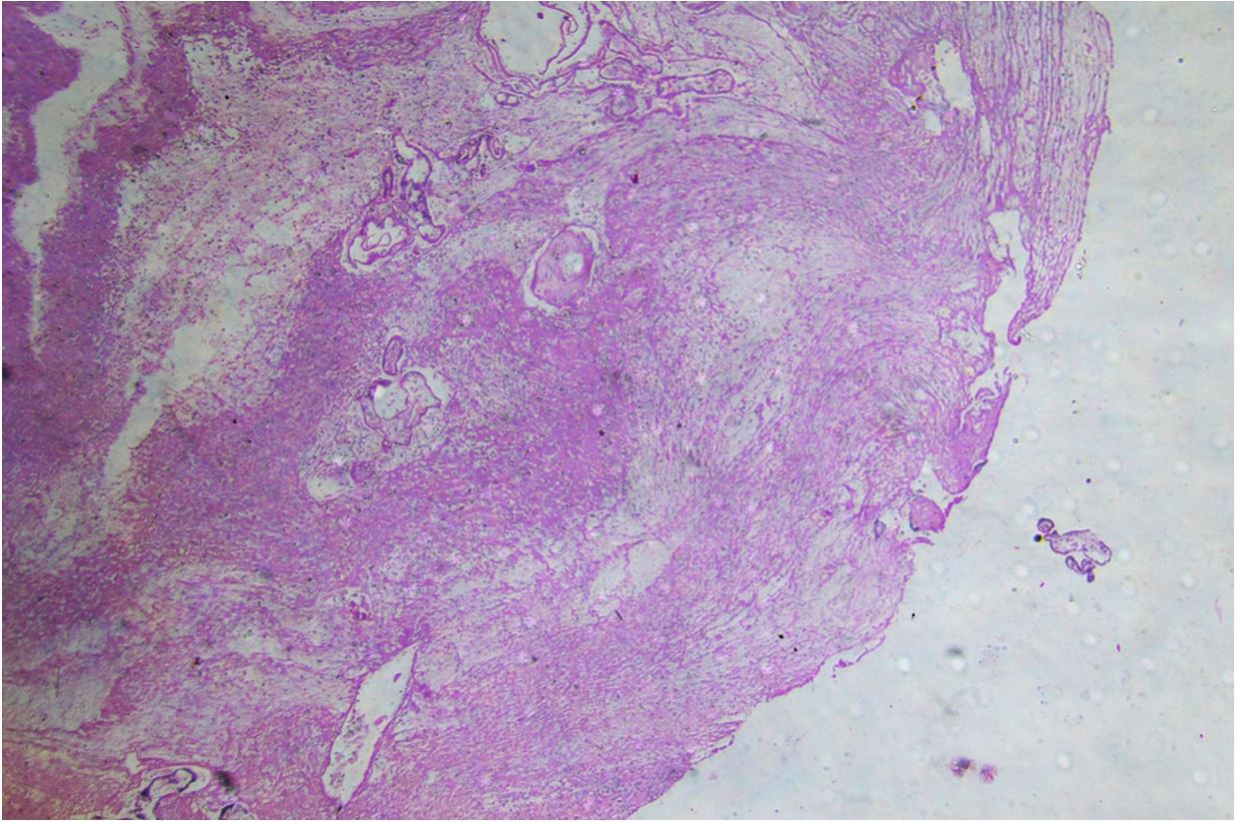
Photomicrograph of the uterus, illustrating chorionic villi invading the full thickness of myometrium to the serosa with evidence of rupture. (H&E, x10).

Histological examination of the margins of the perforated uterine wall showed chorionic villi invading the myometrium and serosa breaching the uterine wall (Figure 2).

Based on gross findings at autopsy and a histopathological examination (Figure 2), we concluded that the patient died due to “hemorrhagic shock due to rupture of previous uterine LSCS scar in a case of placenta accreta presenting in the early third trimester of pregnancy.”

## DISCUSSION

The common causes of maternal death are postpartum hemorrhage, infections, high blood pressure, and unsafe abortion. Garmi and Salim,^3^ mentioned that placenta accreta occurs in approximately 1 : 1000 deliveries ranging from 0.04% to 0.9%, with maternal morbidity of 60% and mortality up to 7% in pregnant women.^3^ The case highlights the fatal risk of aberrant placentation presenting as a decompensated hypovolemic shock due to uterine rupture. Rupture of the uterus is unpredictable and considered to be the most catastrophic obstetric emergency with high maternal and fetal mortality.^4^ ^,^ ^5^ The common risk factors for rupture uterus reported in the literature are grand multiparity, fetal macrosomia, and malpresentation.^6^ ^-^ ^8^ However, in a study conducted in India, the most common factor was found to be prior cesarean delivery.^9^ The consequences of uterine rupture in a placenta accreta depend on the time of diagnosis and delivery.^10^ The signs and symptoms of uterine rupture are variable. They are largely dependent on gestational age, site, and extent of the uterine defect. The patient may present with abdominal pain, distended abdomen with abnormal uterine contour, easily palpable fetal parts, absent fetal heart activity, maternal tachycardia, or hypovolemic shock.

In this case, placenta previa with features suggestive of a placenta accreta was noted on routine antenatal ultrasound check-up, at 24 weeks of gestation, in a 24-year-old pregnant female (G_5_P_2_L_2_A_2_) with history of two previous cesarean section deliveries. Placenta previa refers to a placenta that overlies or is proximate to internal os of the cervix.^11^ In such a setting of a placenta previa and one or more previous cesarean deliveries, the placenta accreta spectrum’s risk is dramatically increased.^12^ Placenta accreta is defined as abnormal trophoblast invasion of part or the entire placenta into the myometrium.^13^ The cohort study by Robert M Silver et al^14^ suggested that in a woman with placenta previa, the risk of placenta accreta is 3%, 11%, 40%, 61%, and 67% for the first, second, third, fourth, and fifth or more cesarean section deliveries, respectively. Placenta accreta spectrum (PAS) is divided into three categories: *Placenta creta* when villi adhere to the myometrium, *Placenta increta* when villi invade myometrium, and *Placenta percreta* when villi invade the full thickness of myometrium.^15^ The rupture of the uterus due to placenta accreta invading previous cesarean scar is most common in the third trimester due to thinned uterine lower segment and mostly reported during labor pains.^16^ ^-^ ^20^ Esposito et al.,^21^ in a case-control study, found that uterine rupture was four times more common in patients than control subjects when the interpregnancy interval between cesarean delivery and the subsequent pregnancy was less than 6 months. Maternal mortality is also reported in such cases due to severe life-threatening hemorrhage.^14^ ^,^ ^17^ ^-^ ^19^ For a favorable outcome in such cases, ultrasound imaging should be used as a screening modality for placenta accreta spectrum disorders. Further MRI evaluation provides high diagnostic and predictive accuracy in assessing both depth and topography of placental invasion.^22^

## RECOMMENDATIONS

This case report indicates that fatal complications can occur in the early phase of the third trimester or maybe even earlier during pregnancy in the light of previous caesarian section deliveries, making awareness extremely valuable in such high-risk groups.

All patients with previous cesarean scars should be advised to avoid pregnancy for at least one year.^23^ The use of barrier or hormonal contraception may be advised for this purpose. Future pregnancy may require early hospitalization and vigilant monitoring because of the risks involved. Before delivery, all women with placenta previa, and their partners should discuss the delivery modalities and be aware of the complications, especially massive obstetric hemorrhage and possible hysterectomy. Innovative strategies with extensive counseling at community and family levels are needed to address the problems leading to high-risk pregnancies to prevent maternal deaths. Despite the number of case reports on severe complications of abnormal placentation, there is surprisingly a lack of studies investigating the microenvironmental changes in the uterine scar leading to fatal invasive trophoblastic invasion. Further studies need to be conducted on micro-assessment of uterine scars and potential ways of preventing severe maternal and fetal complications in such cases.
